# Combating an invisible enemy: the American military response to global pandemics

**DOI:** 10.1186/s40779-021-00299-3

**Published:** 2021-01-25

**Authors:** Lauren K. Dutton, Peter C. Rhee, Alexander Y. Shin, Richard J. Ehrlichman, Richard J. Shemin

**Affiliations:** 1 Department of Orthopedic Surgery, Mayo Clinic, 200 1st Street SW, Rochester, MN 55905 USA; 2 Navy Medicine Professional Development Center, Bethesda, MD 20889 USA; 3 United States Air Force Reserve, the 60th Ops Squadron, Travis AFB, CA 94535 USA; 4 Division of Plastic and Reconstructive Surgery, Massachusetts General Hospital, Boston, MA 02114 USA; 5 Massachusetts National Guard, United States Army, Hanscom AFB, MA 01731 USA; 6 Division of Cardiac Surgery, Department of Surgery, Cardiovascular Center at UCLA, David Geffen School of Medicine at UCLA, Los Angeles, CA 90095 USA

**Keywords:** Pandemic, Spanish influenza, COVID-19, Military, Influenza

## Abstract

The present moment is not the first time that America has found itself at war with a pathogen during a time of international conflict. Between crowded barracks at home and trenches abroad, wartime conditions helped enable the spread of influenza in the fall of 1918 during World War I such that an estimated 20–40% of U.S. military members were infected. While the coronavirus disease 2019 (COVID-19) pandemic is unparalleled for most of today’s population, it is essential to not view it as unprecedented lest the lessons of past pandemics and their effect on the American military be forgotten. This article provides a historical perspective on the effect of the most notable antecedent pandemic, the Spanish Influenza epidemic, on American forces with the goal of understanding the interrelationship of global pandemics and the military, highlighting the unique challenges of the current pandemic, and examining how the American military has fought back against pandemics both at home and abroad, both 100 years ago and today.

## Background

### Coronavirus disease 2019 (COVID-19) pandemic

Following its initial detection in December 2019, coronavirus disease 2019 (COVID-19) spread rapidly across the planet to the extent that social distancing quickly became the “new normal” and droves of citizens across the globe found themselves confined to their homes. In an effort to protect Americans and prevent the spread of disease, millions of individuals were ordered to work from home, travel advisories and bans were set in place across the country and the world, elective medical care was essentially halted, the Olympic Games were postponed, and financial markets dropped precipitously. Indeed, one would be hard-pressed to find an individual or institution whose existence was not substantially altered by this pandemic.

Even as many of the world’s activities and plans were cancelled in accordance with the principle of social distancing, there was one group that remained at work with no plans to return home in the foreseeable future: the soldiers, sailors, airmen, and Marines deployed across the globe. For these members of the Armed forces, the often crowded and austere conditions inherent to deployment frequently also rendered substantial social distancing infeasible. While many other countries forming the U.S. coalition including the United Kingdom, Australia, New Zealand, Italy, Canada, France, Spain, and the Netherlands have recalled some of their troops in response to COVID-19 [1–6], it is estimated that between 60,000 to 70,000 American service members remain deployed in the Middle East [7], with many of their deployments extended in accordance with a 60-day Overseas Stop Movement Order put in place in an effort to prevent the spread of COVID-19 [8]. The first active-duty military case of COVID-19 was reported in a 23-year-old American soldier stationed in South Korea in late February 2020 who resided off base; days later, his wife also tested positive [9, 10].

As of December 23, 2020, the number of military patients that had tested positive for COVID-19 was 101,236 [11]. The first U.S. service member to succumb to the disease was a 57-year-old Army National Guardsman who died on March 28, 2020, after having been hospitalized 7 days prior [12, 13]. As of December 23, 2020, 14 U.S. service members have died [11]. Of the more than 4000 U.S. Navy sailors aboard the USS Theodore Roosevelt, 23 had reportedly tested positive for COVID-19 as of March 26, 2020; this number had increased to “dozens” within 1 week and eventually reached over 1100 cases by April 30, 2020, with one sailor having succumbed to the disease [14–16]. In seeing the rapid spread of the disease across the ship, its then-commanding officer, Capt. Brett E. Crozier, requested that 90% of the ship’s crew be moved into isolation on Guam. This action was deemed necessary by Crozier because of the realities of life aboard an aircraft carrier that rendered attempts to prevent the spread of disease near impossible, including shared sleeping quarters, group mealtimes, and tasks that necessitate the close interaction and proximity of service members [17]. The propensity of this disease to rapidly spread across an aircraft carrier and the need to protect its sailors was likewise recognized by the leadership of the Harry S. Truman Strike Group, which made the decision to extend its deployment in order to safeguard its crew while maintaining its warfighting capability [18].

During this extraordinary period of uncertainty for our planet and our country, the military was rightly called upon to assist in the relief efforts across the country as our hospitals and resources became outpaced by this disease. The American Navy hospital ships USNS Mercy (T-AH-19) and Comfort (T-AH-20) were mobilized to fight this pandemic in Los Angeles and New York City, respectively. While their mission was initially to care for non-COVID patients in order to free up local hospitals to use their full capacity, staff, and resources to treat COVID patients [19], the role of the Comfort later evolved to caring for COVID-positive patients [20]. Likewise, over 40,000 Air and Army National Guard troops were called up to support the pandemic crisis response at the direction of their state governors [21].

While the mobilization of these troops to fight this pandemic has been an appropriately highlighted and visible component of the relief effort for the war at home, it is critical that American citizens are aware that thousands of our service members remain on the front lines of the war on terror, prioritizing the protection of the American people as part of Operation Inherent Resolve and Operation Freedom’s Sentinel. While thankfully no outbreaks amongst American troops deployed to the Middle East have been reported as of the time at this writing, the lack of a robust hospital system capable of caring for a potentially large number of critically ill troops provides an additional daunting challenge in an already austere environment. Indeed, the tiered Joint Trauma System (JTS) of medical facilities downrange is designed to triage and stabilize battlefield injuries, not necessarily to provide potentially long-term respiratory support to large acute volumes of patients. Recent data from the Center for Disease Control indicate that 13.8% of COVID-19 cases occur in patients aged 20–29 with a 3.7% hospitalization rate and 16.3% of cases occur in patients aged 30–39 with a 5.9% hospitalization rate [22]. While these hospitalization rates are relatively low for military-aged men and women, they are substantial enough to indicate that the risk of severe infection exists for this younger population.

The present state of affairs evokes the global pandemic that occurred just over 100 years prior and was likewise intimately interconnected with an intercontinental military conflict. The Spanish Influenza epidemic of 1918 spread across the globe in the fall of 1918 during what would become the height of the American military involvement in World War I in which 20–40% of U.S. Army and Navy service members were estimated to have been infected [23]. In turn, the influenza pandemic profoundly affected the trajectory of the war itself on account of the loss of life it exacted off the battlefield. Between fatalities from the pandemic in Europe and training camps in the United States, over 56,000 American soldiers are estimated to have died from the disease in 1918 as compared to 53,402 killed in combat [23, 24] and the influenza pandemic is believed to have killed more than 50 million people overall [25] (Table 1). In a similar manner, the typhoid fever epidemic of 1898 during the Spanish - American War led to a higher number of American military fatalities than did combat casualties [26]. While the majority of service members, particularly those who are deployed, are young, fit, and healthy, these wars taught us that even young, healthy soldiers are not immune to the spread of viruses sufficiently virulent to cause a pandemic, particularly when combined with close living quarters, harsh environmental exposure, and increased physical and emotional stress that can decrease even a healthy individual’s immunity.

While the COVID-19 pandemic may be unparalleled in the lifetimes of the vast majority of our current population, it is essential to not view it as unprecedented lest the lessons of past pandemics and their effect on the American military be forgotten. The purpose of this article is to provide historical perspective on the effect of the most notable antecedent pandemic, the Spanish Flu epidemic in 1918–1919 during World War I, on the American forces with the goal of comparing these global pandemics, examining their effects on the military during a time of ongoing global conflict, and, particularly in the case of the present epidemic, highlighting the efforts of the military to combat this non-traditional enemy.

### The Spanish influenza pandemic of 1918–1919

At the peak of American military involvement in World War I in the fall of 1918, the United States Army and Navy found themselves at war not only with Germany, but also with an invisible foe that would ultimately take more American lives than the conflict itself: influenza [23]. In her detailed account of the interconnection between the Great War and disease, military medical historian Carol R. Byerly recalls that the inextricably intertwined relationship between these two entities can be traced back to the Spanish and Portuguese conquistadores of the fifteenth century who brought with them the smallpox, flu, and typhus that plagued the New World [23]. As the United States prepared to enter the war in 1917, Navy Surgeon General William C. Braisted expressed confidence that scientific advances leading to the ability to maintain proper ventilation and heating of battleships, distill fresh water from salt water, and understand and prevent scurvy had contributed to the health and wellbeing of the fleet [27]. Moreover, in accordance with the successful development of vaccines, he remarked, “Infectious diseases that formerly carried off their thousands, such as yellow fever, typhus, cholera, and typhoid have all yielded to our modern knowledge of their causes and our consequent logical measures taken for their prevention.” Little could he have known, however, that one covert enemy would prove to be even more lethal than the German army awaiting the American service members as they prepared for combat. Ultimately, more than 26% of Army soldiers were infected with influenza, which amounted to more than one million men [23]. Just as the conditions of training for and fighting battles lent themselves to the rapid spread of the disease, so too did the pandemic evolve to severely compromise the ability of the troops to wage war. In turn, the war became a catalyst for the rapid spread of the disease across continents. As Nobel laureate virologist Frank Macfarlane Burnet concluded, the spread of the 1918 influenza pandemic most likely began in the United States and was “intimately related to war conditions and especially the arrival of American troops in France” [28].

In 1918, naval medical facilities around the world admitted 121,225 Navy and Marine Corps patients with influenza. Of these patients, it is estimated that between 4158 and 5027 sailors succumbed to the virus [23, 29]. At the time, Navy medical personnel understood the importance of maintaining hygiene and sanitation standards to reduce the risk of contagion, even encouraging sailors to refrain from “promiscuous spitting” [29]. As noted by the Surgeon General of the Navy in his annual report to the Secretary of the Navy, “The methods of handling men in respect to their health have improved steadily as time and experience rendered evident an increasing number of officers and men the necessity for measures only fully appreciated at first by the few” [30]. To this end, practices such as frequent handwashing, avoidance of crowding, and isolation of sick patients were also encouraged. Both at home and abroad, however, the acknowledged medical advice to avoid crowding found itself at odds with the needs of the military to continue to recruit, train, transport, and shelter war fighters during World War I.

## Historical perspectives: then and now

### The response on American shores

#### Training camps

The Army began training recruits in the fall of 1917 at 32 training camps, each of which was home to between 25,000 and 55,000 troops [23]. Recruits often found themselves aboard packed trains for days to reach their training camp destinations [29]. As noted in the Annual Report of the Secretary of the Navy in 1919, “The conditions under which drafts are moved by rail, more especially for long distances, almost always involve such predisposing influences as overcrowding, bad ventilation, interrupted sleep, and irregular meals.” [30] In an attempt to prevent the spread of disease, “sneeze screens” were installed in open-bay barracks and tents, and special barracks were designated to isolate symptomatic recruits from healthy ones [29]. While the Marine Corps stopped accepting volunteers in September 1918, as the pandemic was being discovered, they did train 15,000 draftees [29]. As noted by Capt. (ret) Thomas L. Snyder, the executive director of the Society for the History of Navy Medicine, “The troops…caught the virus in training camps, where it spread readily among soldiers living crowded in barracks and being stressed by the rigors of military training” [31].

In response to COVID-19, the process of recruiting and training Armed forces recruits was substantially altered. In late March 2020, more than 20 patients tested positive for COVID-19 at Marine Corps Recruiting Depot (MCRD) Parris Island in Beaufort, S.C., prompting the Marine Corps to temporarily halt new shipments of recruits there [32]. Upon restarting recruit training, recruits were shipped directly to their follow-on combat training instead of returning home on leave in between in order to mitigate the potential spread of the disease amongst the ranks [33]. The Army closed its recruiting stations and transitioned to “virtual recruiting” in March 2020 [34]. After recruits across basic training camps in the Navy, Army, and Air Force also tested positive for COVID-19 in spite of multiple measures established to combat the spread of the disease including staff lock-downs and screening programs [35–37], Secretary of Defense Mark T. Esper issued a memorandum calling for a pause in recruit training for 2 weeks as of April 6, 2020; this temporary suspension was subsequently lifted on April 20, 2020 [38, 39].

#### Military barracks and the response of the medical corps

As the United States prepared to enter the war in 1917, the military grew from 378,000 service members in April 1917 to more than 4.7 million by the war’s end following the establishment of a draft [23]. In order to train and equip these soldiers and sailors, military installations including training camps, arsenals, supply depots, and air fields emerged in every state across the country. As written by historian Barry [40] in *The Great Influenza*, it is theorized that the Spanish Flu pandemic may have begun in Haskell County, Kansas, and then spread rapidly beyond Kansas state lines by way of one such military camp located within Fort Riley. On March 4, 1918, a private at Camp Funston working as a cook reported to sick call with influenza; within 3 weeks, more than 1100 soldiers were sufficiently ill from the disease to require hospitalization, thousands more were treated at infirmaries, and 38 men died [40]. Countless infected soldiers who had been housed at Fort Riley then shipped out to Europe, bringing with them this lethal virus that continued to mutate [41].

Focused on keeping as many soldiers healthy and deployment-ready as possible, the Army Medical Department, which consisted of 30,500 medical officers [42], educated their soldiers regarding the importance of sanitation, good hygiene, and the avoidance of crowding [23]. The number of commissioned officers in the Army Medical Department increased from 833 in April 1917 to 30,591 in November 1918 [42]. The Department of the Navy, meanwhile, had approximately 3000 medical officers [30]. In total, this led to nearly 30% of American physicians being engaged in military service at the time of World War I, thereby creating voids in medical care for the civilian population across the country [23, 43]. To supplement the need for physicians, civilian contract surgeons were employed to augment the Medical Corps; the number of civilian employees in the Medical Department increased from 450 in April 1917 to over 10,500 in November 1918 [42]. Meticulous record-keeping was emphasized in order to track the disease and compare rates of infection with that of civilians, previous conflicts, and other armed forces [23]. Even as they grew their forces, however, there was little even the most skilled and knowledgeable military physicians could do to combat the virus other than provide supportive and comfort measures to their ailing patients and attempt to keep them warm, rested, fed, and hydrated. By the most conservative estimates, influenza infected over one million soldiers and took the lives of almost 30,000 before even they arrived in theater [23, 24]. In an attempt to better understand the disease that was ravaging their armed forces and the world at large, they ran what tests they could and performed autopsies [23].

In today’s fight against COVID-19, in addition to the calling up of Reserve forces to augment civilian and field hospitals across the country as described herein, the military’s medical school, the Uniformed Services University of the Health Science, graduated its students early to allow them to join the fight against the pandemic [44]. Other civilian medical schools also followed suit.

**Fig. 1:**
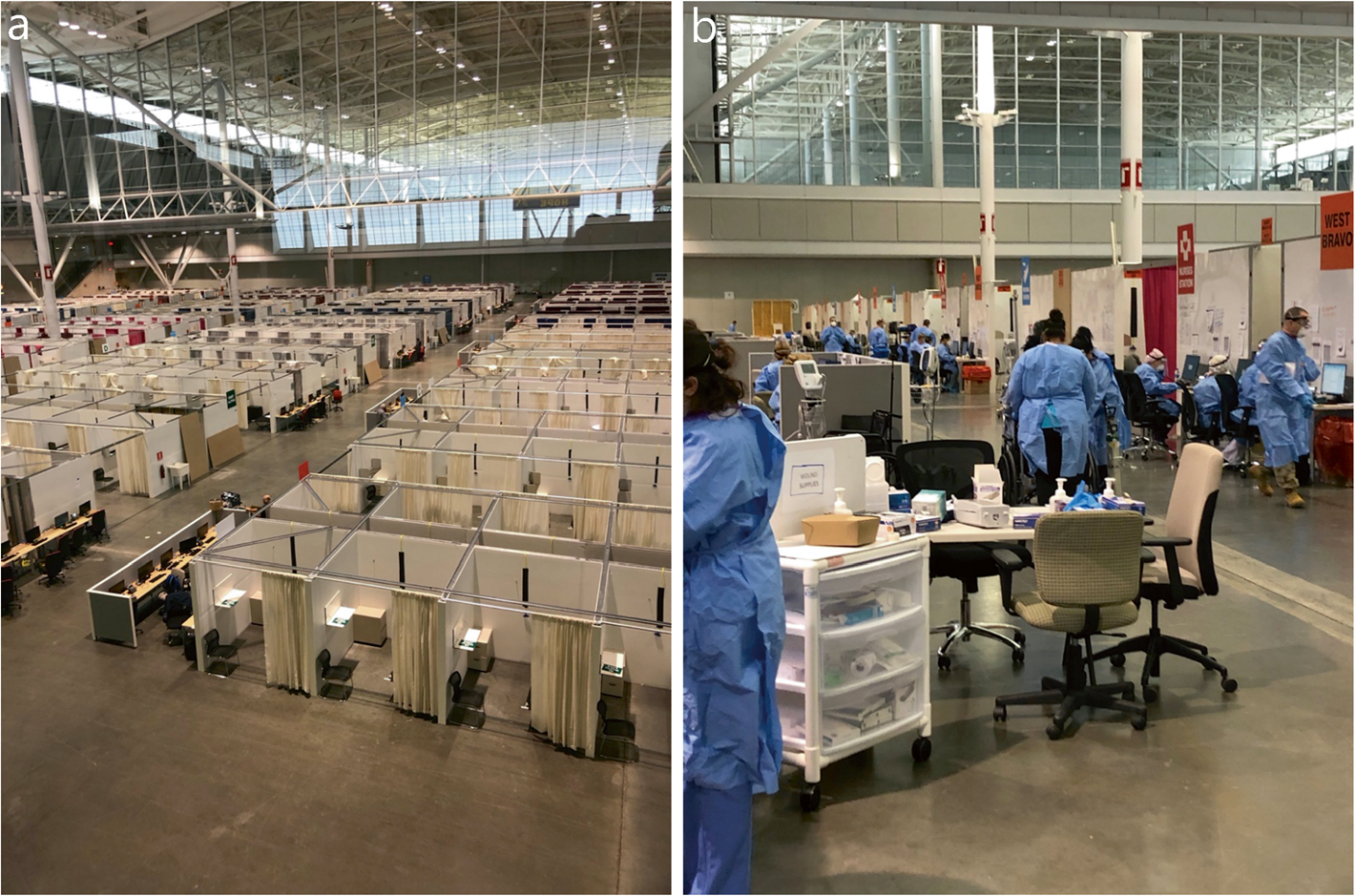
With the assistance of members of U.S. Army Urban Augmentation Medical Task Forces (UAMTFs), the Boston Convention and Exhibition Center was transformed into a field hospital for treatment of COVID-19 patients, complete with patient rooms and nursing stations (**a**). The facility, called Boston Hope Medical Center, began accepting and treating patients in April 2020 (**b**). (Photos courtesy of COL Richard J. Ehrlichman, M.D.)

At a time where the Military Health System (MHS) reform seeks to eliminate up to 18,000 military medical positions across the Armed Forces with the goal of bolstering warfighter readiness [45], the 2020 pandemic has highlighted perhaps the greatest strength of military medicine: the ability to swiftly and effectively provide urgent and emergent medical care to a vast number of patients with scarce resources in non-hospital settings. Examples abound of how tens of thousands of National Guardsmen and Reservists were activated across the country, from the South Carolina National Guardsmen transporting personal protective equipment (PPE) across the state to the Connecticut National Guard building shelters with electricity for housing COVID-19 patients [46]. The Army Corps of Engineers constructed a fully operational 2910-bed temporary hospital at the Javits Convention Center in New York City that was augmented by Army Urban Augmentation Medical Task Forces (UAMTFs) and a Navy Expeditionary Medical Facility [47, 48], and members of the 56th Stryker Brigade Combat Team and the 111th Attack Wing supported the Pennsylvania National Guard in creating a 250-patient medical station as well as community testing site [46]. In Massachusetts and New Jersey, UAMTFs combined forces with civilian hospitals to augment the multidisciplinary medical staff in caring for COVID patients at convention centers that have been converted into field hospitals (Fig. 1). In light of the particularly devastating effect of the pandemic on New York City, some health care providers even provided patient care directly in civilian hospitals to augment their medical staff [49]. Across the country, over 40,000 National Guard members were called up to aid in the relief effort by running COVID testing and alternate care sites, distributing meals and personal protective equipment, and manning food banks [46, 50].

**Table 1:** A comparison between 1918 influenza pandemic and COVID-19 pandemic

	1918 influenza pandemic	COVID-19 pandemic
Causative virus	Influenza A (H1N1)	Severe acute respiratory syndrome coronavirus-2 (SARS-CoV-2)
Average incubation Period [70] (mean, range)	2 days (1–4 days)	5 days (2–14 days) [71–73]
Number of cases worldwide (estimated)	500 million [74]	80.6 million (as of 27 December 2020) [75]
Number of deaths worldwide (est.)	50 million [76]	1.8 million (as of 27 December 2020) [75]
Fatality rate (est.)	2–3% [77]	2.2% (calculated from JHU data: # of deaths worldwide/# of cases worldwide) – 4% [73, 75]
Number of U.S. service members infected (est.)	> 1 million [23]	101,236 (as of 23 December 2020) [11]
Number of deaths among U.S. military (est.)	45,000 [24] to > 56,000 [78]	14 (as of 23 December 2020) [11]
Strategies implemented for spread limitation among military members	Hand hygiene, closing of common spaces, limitation of liberty, sneeze screens	Quarantines/restriction of movement (ROM), stop movement order, de-crowding of ships, social distancing, universal mask wear
Contributions of U.S. military to combat virus	Hospital ships, augmentation of Army Medical Department, post-war influenza research and pandemic preparation efforts	Hospital ships, calling up of reserve forces/National guard/UAMTRs to augment civilian medical care and relief efforts, COVID-19 vaccine development

*UAMTFs* U.S. Army Urban Augmentation Medical Task Forces

#### U.S. Navy hospital ships

The U.S. Navy hospital ships, the Comfort (AH-3) and the Mercy (AH-4), served a dual role during the World War I Spanish Influenza pandemic. In addition to each being briefly deployed to New York to care for overflow patients from land-based hospitals, these “floating hospitals” combined with the USS Solace (AH-2) to cross the Atlantic Ocean and transport thousands of wounded and sick soldiers back to America from Europe [51].

In March 2020, both the USNS Comfort and USNS Mercy were deployed to New York and Los Angeles, respectively, in response to the COVID-19 pandemic. While the initial mission of these hospital ships was to provide free medical and surgical care to non-COVID patients to offload land-based hospitals, in response to the evolving needs of the New York City patient population the USNS Comfort later adapted its mission to accepting COVID-positive patients. This ship is equipped with 100 beds with ventilators, with a goal of ultimately allocating 500 of its 1000 beds for the treatment of severe cases of coronavirus [20, 52]. In addition to the challenges of adapting the Comfort to care for a large number of COVID-positive patients, the ship was also forced to contend with the inherent risk of its own sailors becoming infected as they cared for others and lived in the relatively crowded conditions inherent to a ship. After at least one crewmember on the Comfort tested positive for COVID-19 [53], the Navy employed principles of isolation and quarantine to mitigate the spread of the disease, in addition to taking the extra precaution of bussing roughly 500 of the medical staff to a hotel in between shifts to decrease crowding and allow for proper rest and hygiene [52].

#### Medical innovations by U.S. service members

The military’s contribution to the fight against the influenza pandemic did not come to an end along with the war itself. In 1934, Capt. Albert P. Krueger, a bacteriologist and expert in respiratory diseases who himself had lived through the influenza pandemic, organized the Naval Reserve Laboratory Research Unit No. 1. In the coming years, this lab made tremendous progress on a series of projects focused on influenza, including the development of a detection technique for influenza viruses, preparation of emergency stocks of influenza virus vaccines, the investigation of natural immunity against the influenza virus, and the execution of a study on airborne infections at Navy and Marine Corps shore stations [54, 55].

As of the time of this writing, the Walter Reed Army Institute of Research (WRAIR) is emerging as a leader in the search for a coronavirus vaccine, which per Army Brig. Gen. Michael J. Talley, the commander of U.S. Army Medical Research and Development Command (USAMRDC) and Fort Detrick, which is expected to begin human trials later in fall 2020 [56–58]. As Col. Robert J. O’Connell, deputy commander of WRAIR, stated, “WRAIR is made for circumstances like this. This is why we exist, to be able to respond when there are crises that require research and development to provide solutions for the war fighter” [59].

### The response abroad

#### Transportation to and from theater

As noted by many historians, the most significant contribution of the U.S. Navy to World War I was the convoy system. In addition to destroyers, as many as 24 cruisers accompanied approximately 45 American troopships as well as Allied and neutral commercial vessels as they crossed the Atlantic to and from theater [31]. Vice Adm. Albert Gleaves, commander of Atlantic Convoy Operations between 1917 and 1919, reported that in spite of taking strict hygiene precautions, 8.8% of Army troops transported during the pandemic became ill, which was a similar reported rate for the Navy sailors on the ship [29]. As noted by Gleaves*,* “Although every effort was made to eliminate sick troops at the gangway, it was inevitable that large numbers of incipient cases were taken on board, and naturally the crowded berthing spaces favored contagion” [60]. A total of 789 deaths during transport across the Atlantic were recorded, thereby necessitating many burials at sea [60]. Furthermore, the trans-Atlantic nature of the war itself provided fertile grounds for the rampant spread of the disease.

In modern warfare, U.S. troops are typically transported to and from theater via a combination of chartered and military aircraft. Effective March 13, 2020, the Department of Defense issued an overseas stop movement in response to COVID-19. This order was originally slated to be in effect for 60 days but was subsequently extended with exemptions made for deployments, re-deployments, and basic training [8, 39]. In accordance with this initial order, an estimated 90,000 service members slated to deploy or re-deploy were ordered to remain in place with the intent of curbing the spread of the pandemic [61].

#### U.S. Navy warships

U.S. Navy ships spread across European waters were heavily affected by the pandemic during World War I. One crew member aboard the battleship USS Nevada in Bantry Bay, Ireland, described sickbay lines of at least 50 ft, while the USS Chester in the Mediterranean Sea reported over one hundred men in the sickbay at a given time [29].

The aforementioned memorandum of Capt. Crozier, commanding officer of the USS Theodore Roosevelt, entitled “Request for Assistance in Response to COVID-19 Pandemic,” highlights several of the issues inherent to a warship during a pandemic. He noted that the close contact necessitated by the ship’s space limitations in berthing, dining areas, and restrooms render it nearly impossible to enact the CDC’s guidelines [62]. Indeed, history has shown us that overcrowding is likely one of the greatest drivers of the rapid spread of disease during a viral pandemic. In recognition of the challenges of adequate social distancing on an aircraft carrier, the USS Theodore Roosevelt docked in Guam in March 2020 and more than 4000 crew members went ashore for testing and quarantine, with approximately 800 sailors remaining behind to protect and run the ship [63]. After 55 days, the ship returned to sea in May 2020 with a scaled-back crew of approximately 3000 sailors, temporarily leaving the remaining 1800 sailors on shore in quarantine [64].

#### Deployed service members abroad

As the surgeon general wrote in his 1919 annual report, “When the devastating influenza epidemic was at its height in this country it was suggested by medical officers… that the flow of troops to Europe be temporarily suspended but this period was coincident with … a concentrated effort to increase our fighting force abroad for definite strategic purposes and the military leaders regarded it as essential that the design be prosecuted with unabated vigor” [30]. As the pandemic spread to Europe, allied military installations employed “distancing measures” such as closing YMCAs and limiting liberty. In alignment with Navy tradition, shots of whiskey were offered to sailors and officers alike [29]. In spite of these measures, it is estimated that 15,489 deaths among soldiers deployed to Europe were caused by the influenza pandemic [24].

At the present time, the United States remains engaged in conflicts in Iraq and Afghanistan spanning nearly two decades in the wake of 9/11. Currently, thousands of U.S. troops remain deployed to war zones abroad. In recognition of this need to balance the protection of the U.S. service members with the maintenance of a strong deployed military presence [65, 66], the DoD produced a Practice Management Guide featuring the most up-to-date COVID-related practice guidelines and evidence [67], as well as specific Force Health Protection Guidance to protect the crews transporting suspected COVID patients [68]. On April 5, 2020, Secretary Esper announced that, to the extent practical, all individuals on DoD grounds wear cloth face coverings when unable to maintain a six-foot separation in public spaces [69]. In spite of these measures, significant challenges for deployed service members in the face of this pandemic remain. In addition to the difficulties of implementing social distancing in often densely-populated bases, there is also the challenge of the potential need to provide supportive care to a potentially massive number of troops in a system designed for triage, damage control measures, and prompt evacuation.

## Conclusions

As we look ahead to how we can best protect the most valuable asset of our military—our service members—in the face of this evolving pandemic, it would behoove us to reflect upon and learn from our past. In the words of Spanish philosopher George Santayana, “Those who cannot remember the past are condemned to repeat it.” To this end, as noted by Byerly in her review of the influenza pandemic of 1918–1919, “While the U.S. military had helped to subdue the Germans, the medical profession had failed to conquer an even more deadly, unseen enemy” [23]. She also noted, “The influenza epidemic in the U.S. military therefore provides a cautionary tale about the power of war to change the health environment and the power of disease to influence the conduct of war” [23].

Over a century later, in the midst of another widespread pandemic during a time of war, the military has not only adapted its massive infrastructure in response to this virus, but has also mobilized across the country to provide medical care to our civilian and military populations alike. Indeed, whereas the military became a catalyst for the spread of disease during World War I, it has become one of its most effective combatants during this present pandemic. Let us hope that our advances in science, technology, epidemiology, infectious diseases, and medicine, can lead us to mitigate the toll for those most vulnerable to this virus. Furthermore, let us hope that the military, through its unparalleled network of logistical abilities, medical resources, and defense of freedom throughout the globe that render it both an ally of contagion but also its enemy, will remain as a beacon of hope and help during this time of crisis. Perhaps most critically, while many aspects of this pandemic remain beyond our control and even as our own lives continue to change before our eyes, let us not forget the service members who are courageously risking their lives abroad to defend our country.

## Data Availability

Not applicable.

## Abbreviations

COVID-19: Coronavirus disease 19
UAMTF: Urban Augmentation Medical Task Forces
WRAIR: Walter Reed Army Institute of Research
JTS: Joint Trauma Service
MHS: Military Health System
MCRD: Marine Corps Recruiting Depot
