# Correction to: Combating an invisible enemy: the American military response to global pandemics

**DOI:** 10.1186/s40779-021-00304-9

**Published:** 2021-02-04

**Authors:** Lauren K. Dutton, Peter C. Rhee, Alexander Y. Shin, Richard J. Ehrlichman, Richard J. Shemin

**Affiliations:** 1 Department of Orthopedic Surgery, Mayo Clinic, 200 1st Street SW, Rochester, MN 55905 USA; 2 Navy Medicine Professional Development Center, Bethesda, MD 20889 USA; 3 United States Air Force Reserve, the 60th Ops Squadron, Travis AFB, CA 94535 USA; 4 Division of Plastic and Reconstructive Surgery, Massachusetts General Hospital, Boston, MA 02114 USA; 5 Massachusetts National Guard, United States Army, Hanscom AFB, MA 01731 USA; 6 Department of Surgery, Division of Cardiac Surgery, Cardiovascular Center at UCLA, David Geffen School of Medicine at UCLA, Los Angeles, CA 90095 USA

**Correction to: Mil Med Res 8, 8 (2021)**

**https://doi.org/10.1186/s40779-021-00299-3**

In the original publication of this article [1], the “Disclaimer” section below is missing. The section of ‘Consent for Publication” should be replaced with “Not applicable.” The original publication has been corrected.

Disclaimer:

The views expressed in this paper are those of the authors and do not reflect the official policy or position of the Departments of the Navy, Army, or Air Force, Department of Defense, or the U.S. Government.

Several of the authors are military service members and employees of the U.S. government. This work was prepared as part of their official duties. Title 17 U.S.C. 105 provides that “Copyright protection under this title is not available for any work of the United States Government.” Title 17 U.S.C. 101 defines United States Government work as a work prepared by a military service member or employee of the United States Government as part of that person’s official duties.
